# Knowns and Unknowns about CAR-T Cell Dysfunction

**DOI:** 10.3390/cancers14041078

**Published:** 2022-02-21

**Authors:** Aleksei Titov, Yaroslav Kaminskiy, Irina Ganeeva, Ekaterina Zmievskaya, Aygul Valiullina, Aygul Rakhmatullina, Alexey Petukhov, Regina Miftakhova, Albert Rizvanov, Emil Bulatov

**Affiliations:** 1 Institute of Fundamental Medicine and Biology, Kazan Federal University, 420008 Kazan, Russia; titov.a@blood.ru (A.T.); iaganeeva@kpfu.ru (I.G.); eazmievskaya@kpfu.ru (E.Z.); ajghvaliullina@kpfu.ru (A.V.); ajgulrrahmatullina@kpfu.ru (A.R.); petukhov_av@almazovcentre.ru (A.P.); rermiftahova@kpfu.ru (R.M.); albert.rizvanov@kpfu.ru (A.R.); 2 Laboratory of Transplantation Immunology, National Research Centre for Hematology, 125167 Moscow, Russia; kaminskii.i@blood.ru; 3 Institute of Hematology, Almazov National Medical Research Center, 197341 Saint Petersburg, Russia; 4 Shemyakin-Ovchinnikov Institute of Bioorganic Chemistry, Russian Academy of Sciences, 117997 Moscow, Russia

**Keywords:** chimeric antigen receptor, CAR-T cell, T cell dysfunction, T cell exhaustion, T cell senescence, CAR tonic signaling

## Abstract

**Simple Summary:**

The primary issue of adoptive cell therapy is the poor in vivo persistence. In this context, it is necessary to clarify the fundamental mechanisms of T cell dysfunction. Here we review common dysfunctional states, including exhaustion and senescence, and discuss the challenges associated with phenotypical characterization of these T cell subsets. We overview the heterogeneity among exhausted T cells as well as mechanisms by which T cells get reinvigorated by checkpoint inhibitors. We emphasize that some cancers not responding to such treatment may activate distinct T cell dysfunction programs. Finally, we describe the dysfunction-promoting mechanisms specific for CAR-T cells and the ways to mitigate them.

**Abstract:**

Immunotherapy using chimeric antigen receptor (CAR) T cells is a promising option for cancer treatment. However, T cells and CAR-T cells frequently become dysfunctional in cancer, where numerous evasion mechanisms impair antitumor immunity. Cancer frequently exploits intrinsic T cell dysfunction mechanisms that evolved for the purpose of defending against autoimmunity. T cell exhaustion is the most studied type of T cell dysfunction. It is characterized by impaired proliferation and cytokine secretion and is often misdefined solely by the expression of the inhibitory receptors. Another type of dysfunction is T cell senescence, which occurs when T cells permanently arrest their cell cycle and proliferation while retaining cytotoxic capability. The first section of this review provides a broad overview of T cell dysfunctional states, including exhaustion and senescence; the second section is focused on the impact of T cell dysfunction on the CAR-T therapeutic potential. Finally, we discuss the recent efforts to mitigate CAR-T cell exhaustion, with an emphasis on epigenetic and transcriptional modulation.

## 1. Introduction

Cellular immunity dysfunction is a hallmark of cancer—a result of the disease’s natural evolution toward counteracting the immune pressure. In contrast to dysfunctional T cells, functional T cells perform a range of activities upon stimulation with the cognate antigen. They can (1) expand; (2) secrete effector cytokines (e.g., IL-2, IFNγ, TNFα, perforins, and granzymes) and lyse target cells; (3) survive after removal of antigen stimulation; and (4) do all of the above upon secondary antigen challenge. In this review, a T cell is considered dysfunctional if at least one of these criteria is not met. The most studied dysfunctional state of T cells is exhaustion, which is characterized by the loss of all aforementioned properties of the functional T cells. Nonetheless, T and NK cells exhibit at least two additional well-defined dysfunctional states (senescence and anergy) [1]. Senescent T cells are phenotypically similar to terminal effector T cells in that they do not proliferate in response to antigen stimulation but retain the ability to secrete effector cytokines and kill target cells, in contrast to exhausted T cells (Tex). 

T cell failure in cancer is mediated by multiple mechanisms, with the tumor microenvironment playing a central role in this process. Since the microenvironment varies significantly among different types of cancer, tumor-infiltrating lymphocytes will adopt tumor-specific dysfunctional states that, while similar to T cell exhaustion, possess distinct characteristics. These dysfunctional states are of great interest to the scientific community and require further classification. Additionally, these processes may affect not only naturally developed T cells but also adoptively transferred cell products expanded ex vivo. Although chimeric antigen receptor (CAR) T cells have revolutionized the treatment of hematological malignancies, further advancements are necessary to achieve long-lasting clinical outcomes. Indeed, many relapses of hematological tumors are associated with poor CAR-T cell persistence and are not due to target antigen loss. The low efficacy of CAR-T cells in solid tumors is also a result of intrinsic or tumor-associated T cell dysfunction and the subsequent loss of persistence. Moreover, T cells may be pre-enriched with dysfunctional populations during the manufacturing process. Additional variables affecting cell functionality include genetic modification, such as CAR transduction. 

In this review, we examine mechanisms underlying T cell dysfunction beyond classical CD8^+^ exhaustion during chronic infection. We overview a variety of dysfunctional T cell states, discuss how they may develop in CAR-T cells, and look at the role of CAR-T cell-specific pathways in promoting this dysfunction. We elaborate on the recently discovered tonic CAR signaling and several unanswered questions in this field, such as the optimal composition of the CAR-T cell product and role of the chemotherapeutic pre-treatment in the “fitness” of CAR-T cells. Finally, we also mention the strategies for ameliorating CAR-T cell dysfunction via targeted “point” modification of the distinct inhibitory pathway (i.e., PD-1), as well as broad modulation of the CAR-T cell transcriptional and epigenetic state.

## 2. Dysfunctional States of T Cells

### 2.1. CD8^+^ T Cell Exhaustion

Initially, T cell exhaustion was regarded as a sequential process, with T cells sequentially transitioning first to effector state and ultimately toward dysfunction in response to persistent stimulation with their cognate antigen [2,3]. However, a novel model was proposed that describes exhaustion as an alternative pathway for chronically stimulated T cell differentiation (as opposed to the “conventional” effector/memory differentiation) [4,5,6,7]. According to the model, only T cells with differentiation plasticity (e.g., naïve or memory precursor T cells) can become exhausted (in contrast to terminally differentiated effector cells) [8,9,10].

T cell exhaustion was extensively investigated using a murine chronic lymphocytic choriomeningitis (LCMV) model. Apart from this chronic infection, exhaustion is thought to occur in various settings where antigen exposure persists: in mouse cancer models [4,11,12,13] as well as in human chronic infections (HIV, HCV, and HBV), cancer, and autoimmune diseases [14,15,16,17,18,19,20,21,22,23,24,25,26]. It is widely accepted that Tex exhibit upregulation of inhibitory receptors (IRs), decreased survival (due to lower Bcl-2 and higher Bim expression), poor cytotoxicity, and impaired secretion of cytokines such as IL-2, TNFα, and IFNγ [3,27,28,29,30,31]. Moreover, Tex adopt a distinct epigenetic landscape that is unique enough to consider them a separate T cell lineage [32,33]. There is evidence suggesting that exhaustion is already epigenetically imprinted in T cells on day 5 of chronic LCMV, distinguishing them from memory cells formed on day 5 of acute LCMV [8]. In contrast to memory precursor cells from acute LCMV, these cells expressed increased levels of PD-1 and TOX and expanded inefficiently upon secondary rechallenge. After removal of the chronic antigen stimulation (by transferring Tex into noninfected or acutely infected hosts), some aspects of T cell function could still be restored [11,34,35,36,37]. Despite this, the epigenetic landscape and impaired functionality of terminally Tex remained stable even after checkpoint blockade or antigen removal [6,18,38].

Tex cells are highly heterogeneous [4,5,31,39,40]. CD8^+^ Tex cells are broadly represented by TCF1^+^ Tex progenitors capable of proliferation/self-renewal and TCF1^−^ terminally exhausted cells [39,41]. TCF1 is a transcription factor essential for the development of Tex progenitors and therapeutic response to checkpoint inhibition. More specifically, CD8^+^ Tex were recently found to linearly progress along the following trajectory: Ly108^+^ CD69^+^ (TCF1^+^ PD-1^int^) -> Ly108^+^ CD69^−^ (TCF1^+^ PD-1^int^) -> Ly108^−^ CD69^−^ (TCF1^−^ PD-1^int^) -> Ly108^−^ CD69^+^ (TCF1^−^ PD-1^hi^) [4]. Although described in the chronic LCMV model, these phenotypes also apply to Tex cells from mouse and human melanoma [4,42]. In another study, the authors found a correlation between the epigenetic landscape of Tex and their surface phenotype [37]. TCF1-expressing CD38^low^ CD101^low^ Tex cells possessed a plastic epigenetic state and were able to restore IFNγ and TNFα production, in contrast to CD38^hi^ CD101^hi^ Tex cells that were resistant to reprogramming.

A specific population of terminally differentiated CD8^+^ Tex superior in viral or tumor control was reported [43,44,45]. It resembles short-lived effector cells (SLECs) from acute infection, forms in the presence of IL-21, is distinct from dysfunctional PD-1^hi^ terminally CD8^+^ Tex, has higher cytolytic activity and cytokine secretion, and is characterized by increased expression of CX3CR1, KLRG1, T-bet, and Zeb-2. To support this, the evidence suggests that exhaustion is caused by suboptimal priming of T cells and not persistent antigen per se. For the proper T cell priming, several conditions are required such as strong T cell receptor (TCR) signal, engagement of costimulatory molecules, cytokine-driven inflammation (presence of IL-2 and IL-12, or IFNα/b), and the lack of inhibitory signals [46,47,48,49,50]. If these conditions are met, T cells differentiate into a large pool of cytotoxic short-term effector cells and a small pool of memory cells. When multiple signals are suboptimal, activated T cell differentiation is skewed towards exhaustion. In agreement with this, when CD8^+^ T cells are primed with nonhematopoietic cells or DCs from chronically infected mice, they exhibit greater exhaustion than when primed with appropriate APCs [51,52,53,54].

The clinical relevance of the Tex heterogeneity is supported by the fact that checkpoint inhibition has a differential effect on various exhausted subpopulations. In humans and mice, the effect of at least PD-1/PD-L1 blockade is mediated by TCF1^+^ Tex progenitors capable of self-renewal and replenishment of the TCF1^−^ Tex cell pool [4,6,39,55,56]. Indeed, following PD-1 blockade, CD8^+^ tumor-specific cells do not escape from exhaustion but are largely replaced by other tumor-specific CD8^+^ T cells with a distinct TCR repertoire [57]. For the development of a TCF1^+^ Tex progenitor subset and its continuous recruitment to the tumor site, tumor-draining lymph nodes play the major role [42,58]. In line with this, DCs in those lymph nodes, mediating priming of new T cells are crucial for success of the anti-PD-1 therapy [59,60,61]. Nonetheless, some studies indicate that T cell dysfunction in cancer may differ from the classical exhaustion landscape [11,14]. For example, in sharp contrast to the findings above, a subset of TCF1^+^ dysfunctional lung adenocarcinoma-infiltrating T cells was found to be irresponsive to checkpoint inhibition [14]. Representation of cancer-associated T cell dysfunction as a distinct entity is further supported by comparing its transcriptomic profile with classical exhaustion cases. Although significantly overlapped, the profile included 567 genes with altered expression that was specific for T-cells exhausted in the cancer environment [11,62]. Moreover, a recent study revealed that the dysfunction program might depend on TCR affinity with high-affinity clones adopting a classical exhaustion state, and low-affinity cells developing distinct dysfunction mechanism of “functional inertness” [63]. This observation further supports the broader landscape of cancer-associated dysfunction.

### 2.2. CD4^+^ T Cell Exhaustion

It is well established that CD4^+^ help is necessary for CD8^+^ T cells to function properly [10]. Similar to CD8^+^ T cells, CD4^+^ T cells are prone to exhaustion during chronic infection, with diminished effector functionality [36,62,64,65], impaired TNFα and IFNγ production, and increased PD-1 expression [62,64]. Chronic stimulation can result in a rapid loss of antigen-specific CD4^+^ T cells, which is relevant for human viral infections, such as HIV or HCV [66,67]. The acute self-resolving course of HCV infection was associated with a preserved virus-specific CD4^+^ response [66]. Similar to CD8^+^ T cells, CD4^+^ Tex form a T cell lineage distinct from the well-characterized CD4^+^ T cell phenotypes (Th1, Th2, and Th17) [64]. These cells may promote CD8^+^ T cell exhaustion by producing IL-10 [64,68], but they can also exhibit an upregulated IFN type I response [64,69] and increased production of IL-21 [64]. Interestingly, IL-21 is known to ameliorate CD8^+^ T cell exhaustion. Recently, Chiara et al. demonstrated that melanoma-associated dysfunction in CD4^+^ T cells, characterized by the expression of the known IRs (TIGIT, PD-1, and LAG-3), is IL-27 dependent [62]. This IL-27-driven program was dependent on Blimp-1 and c-MAF and was associated with the expression of several novel dysfunction drivers (PROCR and PDPN). Knocking -out of these genes resulted in improved tumor control. Although both CD4^+^ and CD8^+^ T cells share a common core transcriptional signature of T cell exhaustion, certain IRs (including CTLA4, CD200, and BTLA) and costimulatory molecules (OX40 and ICOS) are biased toward CD4^+^, demonstrating their differential regulation. Finally, similar to CD8^+^ T cell exhaustion, substantial heterogeneity is observed within CD4^+^ Tex cells [64].

These studies demonstrate that while CD4^+^ T cell exhaustion largely resembles that of CD8^+^ T cells, it also possesses distinct regulatory mechanisms that require further elucidation. Though largely ignored by the scientific community, CD4^+^ T cell exhaustion is equally essential and should be considered when developing novel immunotherapies.

### 2.3. How to Combat Exhaustion?

The most well-known and clinically approved approach is the checkpoint blockade (i.e., CTLA inhibition with ipilimumab or PD-L1 inhibition with nivolumab, pembrolizumab, etc.). Importantly, as previously stated, the checkpoint blockade does not reverse exhaustion but rather boosts proliferation of the less differentiated T cell populations, thereby generating an extensive pool of terminally exhausted T cells. While there is currently no way to completely reverse T cell exhaustion (i.e., dedifferentiate Tex into nonexhausted T cell lineages such as memory T cells), the research community has developed numerous strategies to mitigate it and thus improve T cell function. For instance, the engagement of costimulatory molecules is beneficial for T cell antitumor and antiviral activity. T cell functionality is enhanced by the activation of OX40 [70], 4-1BB [71,72], CD27 [73,74], and CD2 costimulatory receptors [15]. Moreover, there is a synergy between the checkpoint blockade and activation of costimulatory molecules that allowed various therapy combinations to achieve significant results. Examples include PD-1 blockade and 4–1BB stimulation in chronic LCMV [75]; PD-1 blockade and OX-40 stimulation in ovarian cancer [76]; CTLA-4 blockade and ICOS or 4–1BB stimulation in melanoma [44,77]; and PD-1 blockade and CD28 stimulation in prostate cancer [78].

Similarly, treatment with inflammatory cytokines, such as IL-2 +/- IL7 [53,79,80], IL-12 [81,82], and IL-21 [45,51,83,84], also improved T cell functionality. Interestingly, these cytokines not only improved T cell functionality but also reinvigorated Tex cells [45,53,81,85]. The efficacy of this strategy was validated in a clinical setting for tumor-infiltrated lymphocyte (TIL)-based therapy [86,87]. Moreover, the PD-1 blockade was synergized with IL-2 in chronic LCMV infection or tumor models [88] and with IL-12 in HBV [85]. 

On the other hand, several soluble factors, including IL-10 [68,89,90,91], IL-35 [92], TGF-β [93], glucocorticoids [13], PGE2 [94], and VEGF-A [25] were shown to promote exhaustion. Along those lines, the PD-1/PD-L1 blockade was synergized with IL-10 or PGE2 inhibition in chronic LCMV [89,90,94,95] and with TGF-β inhibition in melanoma [96]. 

Although multiple strategies were suggested for improving the functionality of Tex, one should keep in mind that exhaustion is epigenetically imprinted and requires complex combinatorial approaches to be truly reversed.

### 2.4. T cell Senescence

Cellular senescence is a stereotypical multicausal process that occurs in a variety of cell types and is characterized by cell cycle arrest. Replicative senescence is telomere-dependent and occurs after a critical number of cell divisions. Telomere-independent senescence, which can be induced by chemotherapy, primarily prevents tumorigenesis upon oncogene activation or DNA damage [97]. Senescent cells exhibit a specific senescence-associated secretory phenotype (SASP) that exerts a paracrine influence on other cells. T cell senescence and T cell exhaustion are distinct processes [2]. They do, however, share some phenotypic features, resulting in terminological confusion and misusage [98,99,100].

Senescent T cells tend to have a CD45RA^+^CD27^−^CD28^−^KLRG1^+^CD57^+^ phenotype, and they lose their proliferative capacity and ability to secrete IL-2, but express cytolytic molecules, IFNγ, and TNFα and are immediately cytotoxic ex vivo [101,102].

T cell senescence is generally associated with the activation of DDR (DNA damage response), which is triggered by telomere erosion, DNA damage caused by reactive oxygen species (ROS), glucose or growth factor deprivation, and cAMP pathway activation [98,103,104]. T cells, in contrast to other cell types, may express telomerase upon activation, conferring on them a certain degree of resistance to replicative senescence [105]. This is demonstrated by the fact that some T cell clones undergo up to 170 population doublings during in vitro culturing [105]. There is mounting evidence that T cell senescence is, in the majority of cases, an active process driven by the telomere-independent p38 pathway [106], and that senescent T cells do not have critically short telomeres [107,108]. The p38-mediated signaling inhibits autophagy, which is necessary for the recycling of damaged mitochondria [108]. Such mitochondria produce excessive ROS leading to DDR, which itself activates p38 in a positive feedback loop [109]. Additionally, p38 inhibits telomerase activity and promotes p16 and p53 activation, which irreversibly arrests the T cell cycle [110,111]. In senescent or glucose-deprived CD4^+^ T cells, sestrin proteins (SESN1/2/3) inhibit mTOR and activate AMPK that upregulates p38, Erk, and JNK via the sMAC complex, and thus contributes to senescence [112,113]. In senescent CD8^+^ T cells, sestrin 2 promotes the expression of the NKG2D/DAPC2 complex via JNK activation, leading to TCR-independent cytotoxicity [114].

Interestingly, IFNα treatment (which drives T cells towards terminal effector differentiation) results in p38 activation, and this potentially accounts for the poor proliferative capacity of the terminal effector population, as well as phenotypic and functional similarities to senescent T cells [110]. Senescent T cells possess their distinct p38-dependent SASP, which differs from the SASP of other cell types and includes TNFα, IL-18, IL-29, CCL5, CCL16, and ADAM28 [115]. It is still not clearly known how this SASP affects other immune cells.

T cells become telomere-independently senescent within the immunosuppressive tumor microenvironment as a result of inhibition by tumor-derived Tregs, γδ T cells, and tumor cells themselves [104,116]. Repeated activation during chronic infection or autoimmune diseases is also associated with T cell senescence [100]. Moreover, tumor-induced senescent T cells have been shown to suppress the activity of other T cells within the tumor microenvironment [104,117]. Although there is increasing evidence that tumors induce T cell senescence as an evasion mechanism, additional studies are required to properly investigate this type of T cell dysfunction and identify options for therapeutic modulation.

**Figure 1:**
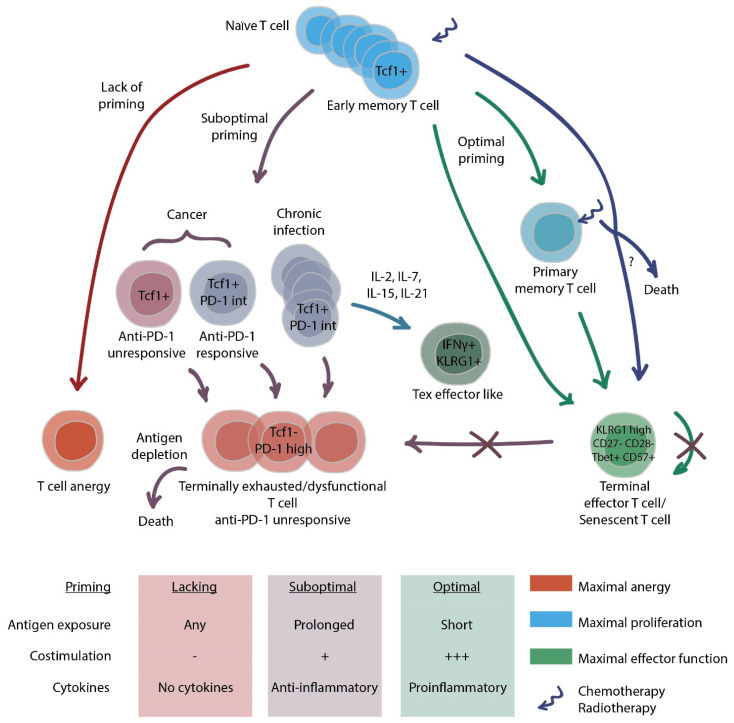
The landscape and evolution of T cell dysfunction. Depending on the priming condition (given at the bottom of the figure), the trajectory of the T cell development may be driven from naïve T cells to (1) anergic T cells; (2) memory T cells; (3) terminal effector T cells; and (4) exhausted T cells (Tex). Memory T cells remain susceptible to exhaustion. Tex cell pool includes progenitor Tex that are primarily responsive to PD-1 blockade and sustain proliferative potential. They give rise to nearly completely dysfunctional terminal Tex or (under certain circumstances) differentiate into highly cytotoxic Tex effector-like state. In some cancers, T cells with a typical progenitor Tex phenotype do not respond to checkpoint inhibition. Both terminally exhausted and effector/senescent T cells are characterized by negligible proliferation; however, the latter demonstrate significant cytotoxicity.

In summary, senescent T cells exhibit increased p38 activation and ROS accumulation, impaired mitochondria activity, downregulated mTOR signaling, and shorter telomeres [108,113,118,119,120]. Despite undergoing cell cycle arrest, these cells retain cytotoxicity and resemble terminal effector T cells. The T cell dysfunction landscape is schematically depicted in Figure 1.

### 2.5. How to Identify Exhausted and Senescent T Cells

Classical exhaustion models are designed in a way that allows exhausted T cells to be readily identifiable (e.g., with MHC-tetramer staining). Nevertheless, there is no established strategy for identification and isolation of exhausted T cells while working with human clinical specimens. Ideally, in order to diagnose T cell exhaustion, one should assess T cell functionality, IR expression, as well as the epigenetic and transcriptional state of a given T cell population. The assessment of T cell functionality (proliferation, cytokine secretion, and cytotoxicity) is of great importance because the surface phenotype (expression of IRs) can be misleading. Indeed, although IR expression often correlates with exhaustion, transient expression of almost all IRs is a hallmark of recently activated effector cells that have no relation to exhaustion [69]. PD-1, for instance, was shown to mark T cells with potent antitumor activity rather than exhausted cells [121]. In light of this uncertainty, large multicolor panels for flow or mass cytometry may be beneficial. One such approach is not only based on the surface and characteristic intracellular Tex markers, such as IRs, TOX, CXCR5, and Eomes, but also includes naïve/memory markers CCR7, CD73, CD127, and TCF-1, which are downregulated in many Tex [122]. However, this strategy is likely to identify only terminally exhausted T cells and not precursor subpopulations. To summarize, despite substantial progress in the development of cytometry exhaustion panels, functional assays remain the most robust and validated method to confirm exhaustion.

Some of the existing approaches for identification of senescent T cells (e.g., based on CD57^+^CD27^low^ and CD28^−^ SA-β-Gal^+^) are yet to be validated [98,100,123]. Particular caution should be exercised when using the CD45RA^+^ marker for terminally differentiated effector cells (TEMRA), since the existence of CD45RA^+^ nonsenescent proliferating memory cells has also been reported [124]. To avoid improper gating, a broad panel of surface markers should be considered that rely on the CD45RA^+^ CD27^−^ CD28^−^ KLRG1^+^ CD57^+^ phenotype of the senescent cells.

## 3. Dysfunctional States of CAR-T Cells

T cells that have been transduced with CAR exhibit similar mechanisms of dysfunction as unmodified T cells. Indeed, CD8^+^ CAR-T cells and TILs isolated from the same tumor-bearing mouse have similar transcriptional and epigenetic profiles [125]. Targeting pathways known to contribute to T cell dysfunction may improve CAR-T cell functionality. This impacts PD-1 [126,127,128,129,130] (including three case reports when CAR-T cells were reinvigorated with anti-PD-L1 treatment [131,132,133]), TIM-3 [129,134,135], CTLA-4 [134], TGF receptor [136,137], and adenosine receptor A2 [138,139]. Other approaches target intracellular signaling mediators [129,140,141,142,143,144,145,146], increase telomerase activity [147], or reverse metabolic inhibition of T cell activity [129,148,149,150]. These are described in more detail in Table 1. 

**Figure 2:**
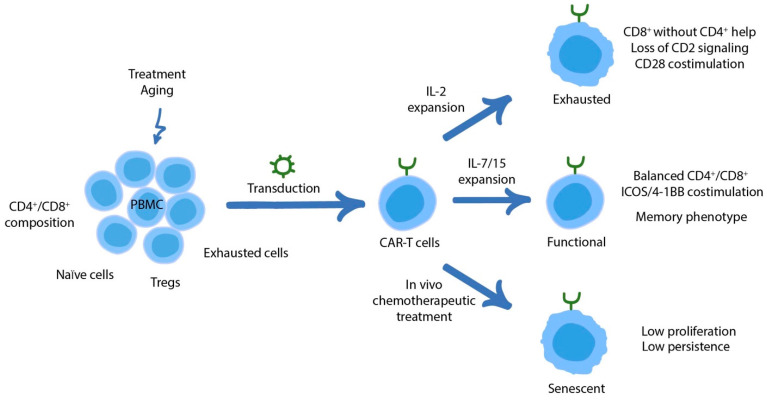
Factors affecting functionality, exhaustion, and senescence of CAR-T cells. PBMCs obtained through apheresis vary substantially in composition and quality. In particular, chemotherapy and older age may result in preemptive T cellular senescence. Apheresis product may be also enriched with Tregs or exhausted cells. These may significantly affect the final CAR-T cell product. At the same time, balanced CD4^+^/CD8^+^ composition and enrichment with naïve cells are known to be beneficial for cell functionality in the clinical setting. Finally, carefully validated CAR design and manufacturing process, e.g., IL-15/IL-7-based expansion, are essential in this context. On the contrary, chemotherapeutic treatment may lead to senescence and poor persistence of CAR-T cells. Several factors are in turn responsible for exhaustion/dysfunction of CAR-T cells in vivo.

Although CAR-T cell-specific exhaustion mechanisms have been recently described, e.g., prolonged tonic CAR signaling, many aspects of such exhaustion remain largely unknown and require further clarification. Does it occur in vivo after adoptive transfer or is it turned on ex vivo during cell expansion? Can it be attributed to the higher affinity of scFv compared to TCR? Is it sustained with the persistence of tumor-associated antigens expressed by healthy tissues (for instance, CD19 on survived tumor B-cells or constantly developing B-cell progenitors)? In addition, the apheresis product composition also plays a critical role, as it may be enriched in dysfunctional/senescent cells or skewed toward a specific phenotype (e.g., exclusively CD8^+^ T cells). This section focuses on CAR-T-specific dysfunction and modulation of their epigenetic and transcriptional profiles, whereas Table 1 summarizes the targeting of common T cell pathways in CAR-T cells. The factors affecting CAR-T cell function in vivo are depicted in Figure 2. 

### 3.1. CAR Signaling as a Driver of Dysfunction and the Road to Its Prevention

A variety of CAR domains and their combinations may differently impact the CAR-T cells dysfunction. Long et al. revealed that GD2-28z CAR-T cells were more prone to exhaustion than GD2-BBz CAR-T cells. This tonic GD2-28z signaling resulted from scFv-mediated CAR clustering that occurred in the absence of an antigen and thus was specific for GD2 CAR-T cells, as opposed to CD19-28z CAR [152]. However, 4-1BB costimulation is not always beneficial. In comparison to 28z CAR, the overexpression of both GD2-BBz or CD19-BBz led to a lower expansion in vitro, higher target cell viability, CAR-T apoptosis, and decreased survival of tumor-bearing mice [153]. The authors found that tonic signaling of overexpressed BBz CAR induces a positive feedback loop, further increasing CAR expression and FasL-dependent apoptosis. This effect was observed both upon stimulation with the CD19^+^ NALM6 leukemia cell line in vivo and without antigen stimulation. Downregulation of BBz CAR expression rescued CAR-T cell function. Notably, an increase in the number of CD4^+^ cells within the total CAR^+^ T cell population upon upregulation of tonic 4-1BB signaling suggests that CD4^+^ CAR-T cells may demonstrate superior resistance to tonic signaling.

Recognizing the crucial role of tonic CAR signaling in CAR-T cell exhaustion prompted an effort to resolve it. Weber et al. recently developed a CAR that requires a stabilizing compound for the assembly and signaling. They showed that transient “resting” of CAR-T cells improved functionality and may help mitigate exhaustion both functionally and epigenetically, even in “aged” CAR-T cells with constant tonic signaling lasting up to 45 days [154]. They demonstrated that dasatinib, an FDA-approved multikinase inhibitor [155], has a similar effect on CAR-T cells underlining its potential clinical application. However, the authors emphasize that their findings contradict those of mouse models in which a late (>14 days) “rescue” of T cells from their cognate antigen did not result in their reinvigoration [5,11].

An alteration of costimulation domains may also contribute to CAR-T cell persistence. Apart from 4-1BB and CD28, another promising costimulator is ICOS. Although CD28 and ICOS are structurally similar and share downstream signaling partners, they have distinct extracellular ligands [156]. In contrast to CD28-based CARs, the ICOS-based ones have been shown to result in dramatically higher persistence of CAR-T cells [157]. Guedan et al. established that this effect depends on the ICOS YMFM-motif [158]. By replacing a single amino acid in CD28 (YMNM->YMFM), they gained persistence levels comparable to ICOS-costimulated CAR-T cells. Notably, the differences were observed 14 days postinfusion, highlighting the long-term effect of YMFM-CD28 or ICOS costimulation on CAR-T cell functionality. ICOS and YMFM-CD28 costimulation drove memory-enriched gene signature and induced Th17-like differentiation of CAR-T cells, which was beneficial in clinical settings (CD19 CAR-T cells) and was associated with durable responses and overall therapeutic efficacy [159,160]. 

A CD2 cell adhesion molecule was previously shown to be an essential costimulator of T cells, preventing exhaustion in an autoimmunity setting, where CD2-dependent costimulation was significantly correlated with a poor prognosis in patients with autoimmune conditions [15]. Accordingly, Majzner et al. observed that the downregulation of CD58 (CD2 ligand) on tumor cells is responsible for significant impairment of CAR-T cell function and leads to markedly reduced progression-free survival in patients with B-cell malignancies [161]. Manipulation of the CD58-CD2 axis may be an attractive option for the generation of improved CAR-T cells, as illustrated by the inclusion of CD2 signaling into the CAR costimulatory module [162].

In summary, T cells with novel CAR designs require extensive investigation to determine their tonic signaling capacity and ability to result in long-lasting cytotoxicity. Multiple costimulation modules (e.g., CD2, ICOS, and YMFM-CD28) may benefit CAR-T cell functionality and help counteract the acquired mechanism of tumor resistance and tumor-dependent CAR-T cell suppression.

### 3.2. Adjusting Regulatory Networks to Counteract T Cell Dysfunction 

The manipulation of a single gene with a defined connection to T cell dysfunction seems an appealing strategy for enhancing the efficacy of CAR-T cells (Table 1). However, inhibitory receptors, for example PD-1, are merely markers of T cell exhaustion and not the cause; thus regulating them is unlikely to reverse this dysfunctional state. Indeed, upon persisting antigen stimulation, PD-1 knockout T cells expanded and functioned well initially; however, later they were severely exhausted, in a much more pronounced manner than wild-type T cells [163]. Moreover, although repeatedly stimulated CD8^+^ T cells were PD-1^+^, they could not be rescued by the blockade of PD-1. Such treatment did not significantly recover antigen-specific T cells compared to the acute infection model [164]. These findings demonstrate that disrupting PD-1 signaling is not always effective and may even be harmful.

Instead of editing the IR gene itself, modifying its enhancer might be more advantageous. Gennert et al. disrupted the exhaustion-associated enhancer of the PD-1 gene in GD2-28z CAR-T cells and discovered a marked reduction in PD-1^high^ CAR-T cells following ex vivo expansion (10 days after CAR transduction) [165]. Preservation of the PD-1 gene enables its expression in nonexhaustion conditions (i.e., following acute stimulation). 

Manipulation of transcriptional networks involved in T cell exhaustion appears to be a more viable strategy than downregulation of specific IRs. For example, in Tex, there is a disbalance between NFAT and AP-1, leading to predominant activation of NFAT-driven exhaustion-associated genes, and not the genes activated by NFAT:AP-1 complexes [166]. Indeed, CAR-T cells overexpressing c-Jun (subunit of AP-1) are more resistant to exhaustion [167]. Overexpression of c-Jun leads to multiple outcomes: increased production of IL-2 and IFNγ in CD19-28z CAR-T cells; IL-2-dependent increase in proliferation of both CD19-28z and CD19-BBz CAR-T cells; reduction in exhaustion markers; and increased efficacy of CAR-T cells in leukemia and osteosarcoma mouse models. Interestingly the authors also observed that c-Jun disrupted AP-1-IRF complexes, allowing the rescue of exhausted CAR-T cells.

Basic leucine zipper transcriptional factor ATF-like (BATF) is another molecule shown to ameliorate T cell exhaustion [168]. Recent research indicated that overexpression of BATF in CAR-T cells also counteracts exhaustion, and this effect depends on BATF interaction with Interferon Regulatory Factor 4 (IRF4) [169]. CD19-28z CD8^+^ CAR-T cells overexpressing transcription factor BATF exhibited increased cytotoxicity, survival, and production of IFNγ, as well as decreased TOX and IR expression. Such cells displayed superior antitumor activity and improved persistence in colon cancer and melanoma mouse models after primary tumor clearance, enabling them to differentiate into memory T cells and protect against a secondary tumor challenge. Interestingly, unlike Lynn et al. [167], this group observed only a limited survival benefit of c-Jun overexpressing CAR-T cells in their melanoma model.

CD8^+^ Tex cells express a high level of the exhaustion-driving transcription factor NR4A and are enriched in NR4A binding DNA motifs [7,37]. The exhaustion-promoting role of NR4A has also been demonstrated in the CAR-T setting [125]. The authors revealed that CD19-28z CD8^+^ CAR-T cells with NR4A triple KO were capable of secreting IFNγ and TNFα upon restimulation and significantly prolonged the cells’ survival in melanoma and colon cancer mouse models expressing CD19. Unlike control cells, NR4A triple KO T cells exhibited the enrichment of NF-kB and AP-1 binding motifs, which may account for improved CAR-T cell functionality. This group also observed a similar improvement in the functionality of CD19-28z CD8^+^ CAR-T cells deficient in both TOX and TOX2 [170]. Indeed, TOX defines the epigenetic landscape of Tex [171,172,173]. The authors proposed a positive feedback loop between NR4A and TOX, potentially explaining the enrichment of NF-kB and AP-1 binding motifs in both NR4A triple KO and double TOX-deficient CAR-T cells. Unlike single KO of TOX2, a single KO of TOX also improved the tumor control. 

Tex activate complex genetic programs orchestrated by a variety of mutually intertwined transcription factors in a specific epigenetic context. Deciphering these programs will enable a combinatorial targeting of transcription factors and, finally, a modifying of epigenetic traits responsible for T cell exhaustion.

### 3.3. The Role of CD4^+^ CAR-T Cells in Counteracting Exhaustion and Overall Therapeutic Efficacy

CD8^+^ CAR-T cells alone were shown to be efficient in treating B-cell malignancies [174,175]. Nevertheless, distinct subsets of CD4^+^ T cells bear helper functions essential for antiviral immunity (Th1 for CD8^+^ T cells and Tfh for B-cell immunity) or autoimmunity (Th17 cells) [15]. Among various predictors of clinical response, the Th17 cytokine secretion profile was identified as important for the clinical efficacy of CAR-T cells [176]. Moreover, in the context of Tex, the loss of CD4^+^ help results in severe exhaustion [2], whereas the transfer of CD4^+^ T cells diminishes exhaustion [10]. Indeed, the design and persistence of CD4^+^ CAR-T cells were critical for the long-lasting function of their CD8^+^ counterparts. Guedan et al. demonstrated that ICOSz CD4^+^ CAR-T cells significantly enhanced the persistence of CD8^+^ CAR-T cells in mice when compared to 28z or BBz CD4^+^ CAR-T cells [157].

Further supporting their importance, CD4^+^ CAR-T cells demonstrated similar killing activity to CD8^+^ in vitro and in vivo, in addition to their helper functions [177,178,179,180]. Although CD4^+^ CAR-T cells exhibited lower initial granzyme B secretion and tumor killing capabilities, they appear to be less susceptible to exhaustion and apoptosis, in comparison to CD8^+^ subpopulation [177,180,181], especially upon dual stimulation through CAR and cognate TCR [180]. These observations provide a rationale for using CD4^+^ T cells for CAR-T cell product manufacturing. Juno Therapeutics pioneered this approach in the clinic with their CAR-T cell therapy consisting of a 1:1 ratio of CD4^+^:CD8^+^ CAR-T cells. One of them (Breyanzi/lisocabtagene maraleucel) was approved by the FDA in March 2021. Although the direct comparisons of different CAR-T cell products are not available, one can speculate that Juno Therapeutic concept demonstrates equivalent or superior antitumor activity with a lower rate of serious adverse events [182,183,184].

### 3.4. Senescence in CAR-T Cells

Senescent T cells may be enriched in the apheresis product obtained from elderly patients or those pretreated with chemotherapy/total body irradiation. Indeed, Guha et al. recently addressed the differential proliferation ability of CAR-T cells derived from young versus elderly donors. They observed that elderly donors showed (i) decreased viral transduction efficiency, (ii) lower levels of proliferation markers, such as p-Akt, p-Erk, and p-STAT5 in CAR-transduced or untransduced IL-2/IL-15 stimulated T cells, and (iii) lower number of either CD4^+^ and CD8^+^ effector and effector memory cells [185]. The authors observed that the majority of CAR-T cells derived from elderly donors are represented by the CD45RA^+^CD45RO^+^ phenotype that is presumably in the process of transition from naïve to memory cells. This effect is likely explained by the increased proportion of senescent and highly differentiated effector T cells in elderly donors, since their naïve T cells remained functional and responded appropriately to the stimulation [186]. Although such senescent cells possess limited proliferative potential and contribute little to the final therapeutic cell product, they still may affect the quality of other T cells during the manufacturing phase via their SASP and contact-dependent interactions [187].

Therapy-induced senescence of CAR-T cells can occur when they are manufactured from the apheresis product obtained after chemotherapeutic treatment [188,189]. Accordingly, pretreatment with cyclophosphamide/doxorubicin-containing regimens appears to be associated with poorly performing CAR-T cells [190,191], which may indicate cellular senescence. Cytotoxic drugs have an impact on T cell proliferation, blasting, and survival. Cytarabine appeared to be the most toxic chemotherapeutic, whereas cyclophosphamide unexpectedly affected not memory, but naïve T cells that are considered quiescent [191]. In line with that, T cells of postchemotherapy patients are represented mainly by effector and memory populations in adults [192] and to a lesser extent in children [193]..

In summary, we may have observed a substantial increase in highly differentiated and senescent T cells in chemotherapy-treated or elderly patients, resulting in lower ex vivo CAR-T cell transduction and expansion. Yet, it remains unclear whether the naïve T cells depletion is due to the cytotoxic effect suspected by Das et al. [191] or caused by their acquisition of senescent phenotype. Nevertheless, we now see the importance of proper timing for effective adoptive immunotherapy, i.e., we should harvest immune cells earlier in the course of patient treatment before they are significantly affected by chemotherapy.

### 3.5. Clinical Correlations of CAR-T Cell Activity/Dysfunction

Biomarkers predicting the efficacy of CAR-T cell therapy are a hot topic of research [194]. Some prediction strategies take into account tumor burden and patient premorbidity (LDH level or estimation of measurable disease), while others reflect CAR-T cell expansion levels (IL-7 and peak CAR transcript). Many researchers stress the importance of less differentiated T/CAR-T cells with higher proliferative capacity (memory T cells [195], including CD27^+^CD45RO^–^CD8^+^ phenotype [159,196]) or IL17A-producing polyfunctional CD4^+^ T cells [176]. Finally, recent clinical trials indicate that a 1:1 CD4^+^:CD8^+^ ratio of CAR-T cells is clinically beneficial [182,196,197]. Unfortunately, most biomarkers associated with therapy efficacy, such as IL-15, low LDH, or peak CAR-T expansion, also correlate with significant and even fatal toxicities [194,198].

## 4. Concluding Remarks

Nowadays, immunotherapy and adoptive cell transfer have become the standard of care for patients with certain B-cell hematological malignancies and are included in the international treatment guidelines. Nonetheless, the long-term clinical benefits can be observed in half of the patients. Improving the treatment outcomes is the primary goal of clinical immunology worldwide. In this context, the underlying causes and mechanisms of T cell dysfunction require further investigation and clarification. Certain similarities are evident between T cell states observed in a variety of chronic disease settings. Indeed, although not indisputable, PD-1/PD-L1 inhibition is now acknowledged as a reason for TCF1^+^ Tex progenitor proliferation and differentiation into TCF1^−^ terminally exhausted T cells, rather than as a cause of terminal exhaustion reversal.

At the same time, despite their functional and transcriptional similarities, tumor-associated dysfunctional T cells may be distinct from their chronic infection counterparts or even from T cells detected in other cancer types. There is a wide range of closely related dysfunctional T cell subtypes associated with autoimmune disorders, infectious diseases, and various types of cancer, each of which requires a unique therapeutic approach.

Although antigen persistence and abundance have long been considered a critical factor for the generation of Tex, evidence is accumulating that optimal priming with optimal cytokine and costimulatory milieu is essential. In agreement with this, Tex were shown to revive, proliferate, and at least partially restore their functionality during cytokine-induced expansion.

CAR-T cells represent a unique phenomenon in the field of synthetic biology. They are regulated by the same exhaustion pathways as conventional T cells, and their modification may significantly boost the efficacy. We believe that CAR signaling itself is an essential exhaustion driver and requires additional in-depth investigations. The “building blocks” of a CAR molecule, including scFv and costimulatory domains, must be carefully examined in terms of compatibility and differential effect on distinct T cell subsets. For instance, ICOS costimulation substantially enhances functions and persistence of CD4^+^ CAR-T cells, the importance of which was unequivocally demonstrated both in animal models and clinically. Finally, modulation of epigenetic and transcriptional regulators in complex with controllable CAR signaling may reduce exhaustion by providing resistance to epigenetic exhaustion programs, and through a transient interruption of antigen stimulation or tonic CAR signaling. Despite the significant scientific progress in this field, many unknowns, such as modulating the epigenetic landscape of CAR-T cells or their senescence, require further research. 

Overall, we believe that future strategies for counteracting T cell dysfunction may help to reduce the relapse rates and complement the range of currently available immunotherapies.

## Figures and Tables

**Table 1:** Modifications of the known T cell dysfunction pathways in CAR-T cells.

Modified Pathway/Molecule	Molecule Type	Upstream Signaling	Downstream Signaling (Activation or *Inhibition*)	Modification	Tumor Model (Animal, Cell Line) and Observed Effect	CAR Construct, Introduction Method	Reference
A2aR (adenosine receptor A2)	surface molecule	adenosine	cAMP	shRNA knockdown A2aR antagonist (SCH-58261)	in vitro, HeLa no significant reduction in cytotoxic function in presence of the adenosine agonist NECA (5′-(*N*-ethylcarboxamide), higher IL-2 expression in comparison to unmodified cells	mesothelin-BBz, lentiviral	[138]
A2aR (adenosine receptor A2)	surface molecule	adenosine	cAMP	shRNA knockdown anti-PD-1 antibody	in vivo, Ly 5.1 mice + 24JK-HER2+ or E0771-HER2+ increased cytokine production of CD8^+^ and activation of CD8^+^ and CD4^+^ CAR-T, particularly with PD-1 blockade; significant survival advantage in mice	HER2-28z, retroviral	[139]
ADAR1 (adenosine deaminase RNA specific)	cytoplasmic enzyme	IFN Type I	biogenesis of members of the miR-222 family -> ICAM1 expression -> immune resistance	EHNA drug (ADAR1 inhibitor)	in vitro, HPAFII, CFPAC, MiaPaCa2 combination with EHNA did not result in reduced survival of the target cells	MUC-1-28z, retroviral	[129]
AKT	intracellular signal transducer	PI3K	MAPK, *FOXO1*	AKT inhibitor VIII (AKTi)	in vitro, NALM6 AKTi repressed glycolysis; in vivo, NOD.Cg-Prkdcscid Il2rgtm1Wjl/SzJ mice + NALM6 AKTi exposed CAR-T elicit significant increase in animal survival	CD19, retroviral	[146]
Argininosuccinate synthase, ornithine transcarbamylase	cancer-associated enzymes	constitutive	arginine, ornithine	Knockin argininosuccinate synthase (ASS) and/or ornithine transcarbamylase (OTC) enzymes	in vivo, arginine-depleted NOG-SCID mice + GD2+ SKNMC, KELLY, and LAN-1; expression of ASS, OTC, or ASS+OTC enhanced proliferation of CAR-T; derived from multiple human donors, regardless of scFv	GD2-BBz CD33-BBz mesothelin-BBz EGFRvIII-BBz	[149]
Catalase	intracellular enzyme	constitutive	H_2_O_2_	coexpression of catalase with CAR	in vitro, SkoV3-Her2+ reduced oxidative state, both basal and upon activation, enhanced proliferation and preserved target cell lysis in presence of H_2_O_2_	CEA-28z, HER2-29z, retroviral	[150]
CTLA-4	surface molecule	CD80/CD86	SHP-2, PP2A	checkpoint blockade by CAR-T-secreted minibodies (reduced checkpoint inhibitors)	in vivo, NSG mice + D270, Hu08-BBz CAR-T cells also showed enhanced tumor reduction when used in combination with anti-CTLA-4, whereas 2173BBz CAR-T cells did not benefit from CTLA-4 checkpoint blockade	see TIM-3 section	[134]
Cyclooxygenase (COX-1, COX-2)	cytoplasmic enzymes	constitutive/inflammation (NFkB)	prostaglandin E2	celecoxib (specific COX-2 inhibitor) and indomethacin (COX1 and 2 inhibitor)	in vitro, HPAFII and CFPAC cells showed a reduction in survival, MiaPaCa2 cells showed no difference in survival; Celecoxib did not change the efficacy of CAR-T cells	MUC-1-28z, retroviral	[129]
Telomerase	cancer/memory T-cell-associated enzyme	p38; DDR	Restores telomere length	transient delivery of telomerase mRNA	in vitro, Raji prolonged proliferation and inhibited cell senescence; in vivo, NPG/Vst mice + Raji, improved persistence, proliferation, and long-term antitumor effects	CD19-28z, CD19-BBz, lentiviral	[147]
IDO-1 (indoleamine 2,3-dioxygenase)	enzyme with high activity in cancer cells	constitutive	kynurenine	IDO inhibitor (1-methyl-tryptophan)	in vivo, SCID-Beige mice + Raji/Raji-IDO CAR-T inhibited IDO-negative but not IDO-positive tumors growth; IDO inhibitor restored IDO-positive tumor control; tryptophan metabolites inhibited expansion, proliferation, cytotoxicity, cytokine secretion, and increased CAR-T apoptosis; 4-1 BB intracellular domain had no effect on the inhibition	CD19-z/BBz, retroviral	[148]
IL-12, IL-18	cytokines	-	STAT4	inducible single-chain p45-p30 IL-12 (iIL-12); 18-kD IL-18 (iIL-18); constitutive IL-18 and IL-12	in vivo, CEA transgenic C57BL/6 mice + Panc02-CEA^+^ and Rag2/γc/mice + A549 CEA^+^ iIL-18 CAR-T exhibited superior activity against large pancreatic and lung tumors refractory to CAR-T cells without cytokines. IL-18 induced overall change in the immune microenvironment	CEA-28z, retroviral	[151]
mTORC1	intracellular signal transducer	calcineurin/DAPK1	*mTORC2/T-bet*	rapamycin (mTOR inhibitor) expansion with IL-15	in vivo, NSG mice, Raji IL15-expanded CAR-T mediated superior antitumor activity, longer persistence, and significantly greater survival than IL-2-expanded CAR-T; IL2/rapamycin-cultured CAR-T shared phenotypic features with IL-15-CAR-T, suggesting that IL15- mediated reduction of mTORC1 activity is responsible for preserving less differentiated phenotype	CD19 second generation, IL13Rα2 second generation, lentiviral	[145]
PD-1	surface molecule	PD-L1/2	SHP-1, SHP-2	antibodies	in vitro MOLM-14, primary leukemia improved cytokine production and Ki-67 proliferation marker, especially in combinational treatment with PD-1 and TIM-3 antibodies; in vivo, NSG mice + MOLM14 increased durable complete response rate	CD33-BBz, CD123-BBz, lentiviral	[127]
PD-1	surface molecule	PD-L1/2	SHP-1, SHP-2	CRISPR/Cas9 KO	in vivo, NSG mice + CD19+PD-1L+ K562, improved clearance of tumor	CD19-BBz, lentiviral	[128]
PD-1	surface molecule	PD-L1/2	SHP-1, SHP-2	checkpoint blockade by CAR-T-secreted minibodies (reduced checkpoint inhibitors)	see TIM-3 section		[144]
PD-1	surface molecule	PD-L1/2	SHP-1, SHP-2	anti-PD-1 Ab	in vitro, no improvement in the killing of the resistant cell lines (HPAFII, CFPAC), while sensitive cell line (MiaPaCa2) killing was enhanced	MUC-1-28z, retroviral	[129]
PD-1	surface molecule	PD-L1/2	SHP-1, SHP-2	pembrolisumab (anti-PD-1 Ab)	clinical case report, mediastinal B cell lymphoma; remission continuing 12 months posttherapy	CD19-BBz, lentiviral	[131]
PD-1	surface molecule	PD-L1/2	SHP-1, SHP-2	nivolumab (anti-PD-1 Ab)	clinical case report, diffuse large B cell lymphoma short tumor volume reduction, lasting 2 months, followed by progression	CD19-BBz	[132]
PD-1	surface molecule	PD-L1/2	SHP-1, SHP-2	nivolumab (anti-PD-1 Ab)	clinical case report, refractory follicular lymphoma remission lasted for >10 months.	CD19-28z, Axicabtagene ciloleucel (Kymriah^®^), retroviral	[133]
PD-1	surface molecule	PD-L1/2	SHP-1, SHP-2	CRISPR-Cas9 KO	in vitro, PC3; in vivo, NSG mice + NALM6/NALM6-PD-L1+; increased cytotoxicity	PSCA-second generation, lentiviral	[130]
PI3Kδ (phosphatidylinositol-3-kinase p110δ)	intracellular signal transducer	TCR and costimulatory molecules (CD28, 4-1BB, and ICOS)	AKT	Idelalisib and AKT inhibitor VIII (AKTi)	in vivo, NSG mice + M108; Idelalisib-treated CAR-T cells exerted longer tumor control compared to the AKTi-treated; Idelalisib T cells have improved engraftment, persistence, less differentiated phenotype, and transcriptional signature	mesothelin-BBz, lentiviral	[143]
PI3Kδ (phosphatidylinositol-3-kinase p110δ)	intracellular signal transducer	TCR and costimulatory molecules (CD28, 4-1BB, and ICOS)	AKT	PI3K inhibitor LY294002	in vitro, MOLM-13 increased effector molecules expression, less differentiated phenotype, higher cell number	CD33-BBz, MOLM-13 target cells (CD33+), retroviral	[144]
PKA (protein kinase A)	intracellular signal transducer	cAMP	Csk	transduction with regulatory subunit I anchoring disruptor (RIAD)	in vitro, AE17ova, PDA4662 cells, increased cytokine release; in vivo, C57BL/6 (strain CD45.2) + EM-meso^+^ and NSG mice + AE17-meso^+^; adenosine or PGE2 did not affect RIAD CAR-T; higher median reduction in tumor volume	mesothelin-BBz, lentiviral (human), retroviral (mice)	[142]
PP2A (protein phosphatase 2A)	intracellular signal transducer	constitutive	*AKT, mTOR, Erk, CaMKKII/IV*	protein phosphatase 2A (PP2A) inhibitor (LB-100)	in vitro, U251-Luc significantly increased cytotoxicity in a dose-dependent manner; in vivo, NSG mice + U251-Luc, significant increase in CD3+ cells within tumor tissue and more frequent tumor regression	CAIX (carbonic anhydrase IX)-BBz	[141]
PTPN-2 (protein tyrosine phosphatase nonreceptor type 2)	constitutive	c-Src	PTPN2-inhibitor compound 8	siRNA duplexes transient knockdown, CRISPR-Cas9 KO	in vivo, female Ly5.1 B6.SJL-Ptprc a Pepc b/BoyJ, human HER-2 transgenic + E0771-HER-2^+^; improved immune surveillance on spontaneous tumors and after adoptive transfer	murine CAR-T: human HER-2-28z, human CAR-T: human LeY-28z, retroviral	[140]
SHP-1/THEMIS complex	intracellular signal transducer	PD-1; CD19-BBz	CD3ζ	knockdown THEMIS or SHP1 in CD19-BBz- CAR-T cells, shRNA	in vitro, BV173-CD19^+^; increased CAR-CD3ζ; basal phosphorylation	CD19-BBz, CD19-28	[126]
TGF-β receptor II	surface molecule	TGF-β	SMAD2/3/4	knockin (together with CAR), dominant-negative TGF-βRII	in vivo, NSG mice + PC3; increased proliferation, cytokine secretion, resistance to exhaustion, long-term in vivo persistence, induction tumor eradication in aggressive human prostate cancer	PSMA-BBz, lentiviral	[136]
TGF-β receptor II	surface molecule	TGF-β	SMAD2/3/4	CRISPR/Cas9 KO	in vitro, mesothelin^+^ CRL5826 and OVCAR-3, improved killing; in vivo, NPG mice + CRL5826; better controlling tumor growth; TGF-β-RII KO outperform PD-1 KO in efficacy; PD-1 KO complement; TGF-β-RII KO	Mesothelin-28z, lentiviral	[137]
TIGIT	surface molecule	CD155 and CD112	SHP-1; b-arrestin/SHIP1-mediated downstream inhibition of *NF-kB, PI3K and MAPK* pathways	TIGIT-28 chimeric switch receptor (TIGIT exo- + CD28 signaling domain)	in vitro, Raji, JY, 721.221, Nalm6; improved killing and cytokine secretion	CD19-BBz, MSGV1-based, retroviral	[135]
TIM-3	surface molecule	galectin-9	CD45; CD148	checkpoint blockade by CAR-T-secreted minibodies (reduced checkpoint inhibitors)	in vivo, NSG mice + D270; significant inhibition of tumor growth with either 2173BBz or Hu08BBz CAR-T cells and anti-PD-1/anti-TIM-3	anti-IL-13Rα2-BBz (humanized Hu08BBz, murine 2173BBz), lentiviral	[134]
TIM-3	surface molecule	galectin-9	CD45; CD148	Gal-9 blocking Ab	in vitro, HPAFII and CFPAC cells—improvement; MiaPaCa2 cells—no improvement	MUC-1-28z, retroviral	[129]

## List of Abbreviations

A2aR	Adenosine Receptor A2
ADAR1	Adenosine Deaminase RNA Specific
AKTi	AKT inhibitor VIII
ASS	Argininosuccinate synthase
BATF	Basic leucine zipper transcriptional factor ATF-like
CAIX	Carbonic anhydrase IX
CAR	Chimeric Antigen Receptor
CAR-T cell	T cell with Chimeric Antigen Receptor
COX	Cyclooxygenase
DDR	DNA Damage Response
HBV	Hepatitis B Virus
HCV	Hepatitis C Virus
HIV	Human Immunodeficiency Virus
IDO-1	Indoleamine 2,3-dioxygenase
IFN	Interferon
iIL	Inducible interleukin
IL	Interleukin
IR	Inhibitory Receptors
IRF4	Interferon Regulatory Factor 4
KO	Knockout
LCMV	Murine Lymphocytic Choriomeningitis
LDH	Lactate Dehydrogenase
MHC	Main Histocompatibility Complex
OTC	Ornithine transcarbamylase
PI3Kδ	Phosphatidylinositol-3-kinase p110δ
PKA	Protein Kinase A
PP2A	Protein Phosphatase 2A
PTPN-2	Protein tyrosine phosphatase non-receptor type 2
ROS	Reactive Oxygen Species
SASP	Senescence-Associated Secretory Phenotype
shRNA	Small hairpin RNA
SLEC	Short-Lived Effector Cells
TCR	T Cell Receptor
Tex	Exhausted T Cell
TIGIT	T cell immunoreceptor with Ig and ITIM domain
TIL	Tumor Infiltrating Lymphocyte
